# Cardiac Abnormalities in Individuals Aged ≥ 50 Years with Severe Obesity Referred for Bariatric Surgery

**DOI:** 10.1007/s11695-024-07422-y

**Published:** 2024-08-01

**Authors:** Michelle Lobeek, Aniek Peters, Sophie L. van Veldhuisen, Yves G. C. J. America, Michiel Rienstra, Eric J. Hazebroek, Dirk J. van Veldhuisen, Thomas M. Gorter

**Affiliations:** 1 Department of Cardiology, University Medical Center Groningen, University of Groningen, Hanzeplein 1, PO Box 30.001, 9700 RB Groningen, The Netherlands; 2 Department of Surgery, Amsterdam UMC, University of Amsterdam, PO Box 22660, 1100 DD Amsterdam, The Netherlands; 3 Department of Surgery/Vitalys Clinic, Rijnstate Hospital, PO Box 9555, 6800 TA Arnhem, The Netherlands; 4 Department of Cardiology, Rijnstate Hospital, PO Box 9555, 6800 TA Arnhem, The Netherlands; 5 Division of Human Nutrition and Health, Wageningen University & Research, PO Box 9101, 6700 HB Wageningen, The Netherlands

**Keywords:** Bariatic surgery, Obesity in the elderly, Cardiovascular disease, Heart failure, Echocardiography

## Abstract

**Supplementary Information:**

The online version contains supplementary material available at 10.1007/s11695-024-07422-y.

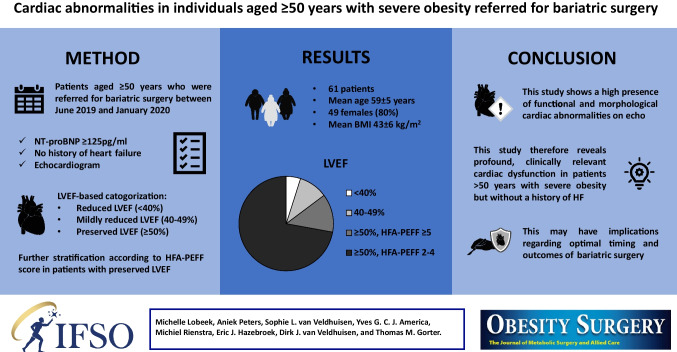

Obesity is one of the most important causes of cardiovascular (CV) disease, mainly heart failure (HF) with a preserved ejection fraction (HFpEF). In particular, patients aged ≥ 50 years with severe obesity are at increased risk for the development of HFpEF. [1] In individuals with severe obesity, bariatric surgery was associated with a reduced risk for the development of CV disease, in particular HF. [2] However, especially individuals aged ≥ 50 years with severe obesity may already have profound subclinical myocardial abnormalities. N-terminal probrain natriuretic peptide (NT-proBNP) is a sensitive marker to detect CV disease. [3] It has been suggested that NT-proBNP also can have clinical relevance as a preoperative screening tool in patients aged ≥ 50 years referred for bariatric surgery. [4] While the number of individuals that is referred for bariatric surgery is increasing, [5] the prevalence of echocardiographic abnormalities in these obese patients remain under represented in the literature. Therefore, the present study investigates the echocardiographic characteristics of individuals aged ≥ 50 years with severe obesity and elevated NT-proBNP levels who were referred for bariatric surgery. In particular, we assess the presence of echocardiographic abnormalities related to HFpEF.

This study is an echocardiographic substudy of an earlier prospective study. [4] Consecutive patients who were referred to the Department of Bariatric Surgery of the Rijnstate Hospital (Arnhem, The Netherlands) between June 2019 and January 2020 were included. Patients aged ≥ 50 years, with NT-proBNP ≥ 125 pg/ml, without a history of HF, meeting the International Federation for the Surgery of Obesity and Metabolic Disorders criteria (i.e. body mass index (BMI) ≥ 35 kg/m^2^ with an obesity-related co-morbidity or BMI ≥ 40 kg/m^2^), and who underwent an echocardiogram due to elevated NT-proBNP levels were included. The study was approved by the Local Ethics Committee. Informed consent was obtained from all individual participants included in the study. All procedures performed in studies involving human participants were in accordance with the ethical standards of the institutional and/or national research committee and with the 1964 Helsinki declaration and its later amendments or comparable ethical standards.

All echocardiographic analyses were performed according to recommendations of the American Society of Echocardiography and are described in detail in the Supplementary Material. [6] Recommended cutoff values for abnormality of each individual echocardiographic measure were used. [6] Patients were categorized based on their left ventricular (LV) ejection fraction (LVEF) into: i) reduced (< 40%), ii) mildly reduced (40–49%), and iii) preserved (≥ 50%) LVEF. Patients with a preserved LVEF were further stratified according to the HFA-PEFF (Heart Failure Association – Pretest Assessment, Echocardiographic and Natriuretic Peptide Score, Functional Testing, Final Etiology) diagnostic algorithm and HFA-PEFF scores for each individual were subsequently calculated. [7].

In total, 61 patients were included. Mean age was 59 ± 5 years, 49 (80%) were female, mean BMI was 43 ± 6 kg/m^2^ and median NT-proBNP was 222 [144–343] pg/ml. Forty-one patients (67%) had hypertension, 5 (8%) had coronary artery disease, 15 (21%) had a history of atrial fibrillation and 15 patients (25%) had type 2 diabetes. Table 1 depicts the echocardiography characteristics of the study population. 26% of the patients had concentric LV remodelling with an increased relative wall thickness. Respectively 39% and 52% of patients had LV diastolic dysfunction, evidenced by a reduced septal and lateral e’. In total, 29% had an increased left atrial volume index, 52% had a reduced LV global longitudinal strain, 52% had an elevated E/e’ and one patient (2%) had increased LV filling pressure with E/e’ > 15. Three patients (5%) had a reduced LVEF, six patients (10%) had a mildly-reduced LVEF and 52 patients (85%) had a preserved LVEF. In the 52 patients with a preserved LVEF, eight patients (15%) had a HFA-PEFF score ≥ 5 points, thus meeting the definite criteria for HFpEF diagnosis. Additionally, 44 patients (85%) had a HFA-PEFF score of 2 to 4 points, suggesting a potential HFpEF diagnosis. Of the 52 patients with a preserved LVEF, 17 (33%) did not have complete data to sum all echocardiographic items of the HFA-PEFF score. Despite this limitation, nine of these patients already had a HFA-PEFF score of 3 or 4 points based on incomplete echocardiographic data.

**Table 1:** Echocardiographic characteristics of the study population

	Mean value	N with abnormal value in all patients (*n* = 61)	N with abnormal value in patients with LVEF ≥ 50% (*n* = 52)
LV ejection fraction, % (*n* = 61)	58 ± 11	9 (15%)	-
LV mass index, g/m^2^ (*n* = 58)	73.8 ± 16.5	5 (9%)	5 (9%)
Relative wall thickness (*n* = 53)	0.41 ± 0.17	14 (26%)	11 (21%)
Septal e’, cm/s (*n* = 61)	7.5 [6.5–8.9]	24 (39%)	21 (34%)
Lateral e’, cm/s (*n* = 60)	9.9 [8.3–11.2]	31 (52%)	27 (45%)
E/e’ (*n* = 60)	9.1 ± 2.4	30 (50%)^a^ 1 (2%)^b^	25 (42%)^a^ 1 (2%)^b^
LA volume index, ml/m^2^ (*n* = 54)	26.7 [22.0–31.0]	11 (20%)^c^ 5 (9%)^d^	8 (15%)^c^ 5 (9%)^d^
LV GLS, % (*n* = 48)	-16.0 [-18.4–4.0]	25 (52%)	19 (40%)
TR regurgitation velocity, m/s (*n* = 12)	2.4 ± 0.1	1 (8%)	1 (8%)

Data are presented as mean ± standard deviation, median [interquartile range] or numbers (percentages). GLS global longitudinal strain

*LA* left atrial, *LV* left ventricular, *TR* tricuspid regurgitation

^a^E/e’ 9–14

^b^E/e’ ≥ 15

^c^LA volume 29–34 ml/m^2^

^d^LA volume ≥ 34 ml/m^2^

In summary, this study showed that a significant number of patients aged ≥ 50 years with severe obesity, who were free of HF, already had profound, cardiac dysfunction mostly related to concentric LV remodelling, LV diastolic dysfunction and reduced LV longitudinal strain. Moreover, this study demonstrated that 15% of the patients already met the HFA-PEFF score criteria, fulfilling the diagnosis of HFpEF. Since HFA-PEFF scores could not be calculated completely in all individuals, the number of patients with echocardiographic abnormalities complying with the diagnosis of HFpEF may even be underestimated in this study. Of note, this was a cross-sectional study without a control group and it was only performed in individuals who were referred for bariatric surgery, which limits generalizability. Nevertheless, this study reveals clinically relevant functional and morphological echocardiographic abnormalities in a substantial number of patients aged ≥ 50 years with severe obesity who were referred for bariatric surgery, but without a history of clinical HF. Further study is needed to investigate whether these findings will have clinical implications regarding the optimal timing of bariatric surgery or in predicting early and late postoperative outcomes.

### Supplementary Information

Below is the link to the electronic supplementary material.

Supplementary file1 (DOCX 41.9 KB)

## Data Availability

The data that support the findings of this study are available from the corresponding author, T.M.G., upon reasonable request.
