# Supplementary material for: Cardiac Abnormalities in Individuals Aged ≥ 50 Years with Severe Obesity Referred for Bariatric Surgery
Source: Obes Surg. 2024 Aug 1;34(9):3513–6. doi: 10.1007/s11695-024-07422-y (PMC11349804; doi:10.1007/s11695-024-07422-y)
Supplement: Supplementary file 1 — Supplementary file1 (DOCX 41.9 KB) [file 11695_2024_7422_MOESM1_ESM.docx]

**SUPPLEMENTARY MATERIAL**

**Echocardiographic analysis**

Transthoracic 2-dimensional echocardiography was performed using Epiq Philips (EPIQ 7 C, hardware and software version 5.02) using standard techniques. HeartModel software was used to measure left ventricular (LV) and left atrial (LA) volumes at end-diastole and at end-systole. Subsequently, left ventricle ejection fraction (LVEF) was measured.

Echocardiographic variables were evaluated in accordance with current American Society of Echocardiography Guidelines.(1) In addition, cutoff values for abnormality of each individual echocardiographic parameter were also chosen according to these recommendations.(1) Increased septal wall thickness was designated as exceeding 10 mm in men and 9 mm in women. Relative wall thickness was considered elevated when >0.42. Abnormal posterior wall thickness was defined as greater than 10 mm for both men and women. LV hypertrophy was diagnosed when the LV mass index exceeded 115 g/m^2^ in men and 95 g/m^2^ in women. Abnormal diastolic tissue velocities of the LV lateral and septal mitral valve annulus were identified as lateral e' velocity less than 10 cm/s and septal e' velocity less than 7 cm/s. An E/e' ratio between 9 and 14 was considered mildly elevated, and was considered abnormal if ≥15. LA volume index was deemed mildly abnormal when between 29 ml/m^2^ and 34 ml/m^2^ and severely abnormal if ≥34 ml/m^2^.

LV deformation was used for the measurement of LV global longitudinal strain (GLS) using TOMTEC-arena software (Tomtec Imaging Systems, Unterschlessheim, Germany). Tracing lines along the endocardial border of the LV were manually placed and LV GLS was measured during one cardiac cycle. Measurements were averaged from the apical 4-chamber, 2-chamber, and 3-chamber views. Using at least two different apical views to calculate LV GLS was acceptable.(2,3) Abnormal LV GLS was defined as > -16%.(4)

**REFERENCES SUPPLEMENTARY MATERIAL**

1. Lang RM, Badano LP, Victor MA, Afilalo J, Armstrong A, Ernande L, et al. Recommendations for cardiac chamber quantification by echocardiography in adults: An update from the American Society of Echocardiography and the European Association of Cardiovascular Imaging. Journal of the American Society of Echocardiography. 2015;28:1-39.e14.

2. Mor-Avi V, Lang RM, Badano LP, Belohlavek M, Cardim NM, Derumeaux G, et al. Current and evolving echocardiographic techniques for the quantitative evaluation of cardiac mechanics: ASE/EAE consensus statement on methodology and indications: Endorsed by the Japanese Society of Echocardiography. Journal of the American Society of Echocardiography. 2011;24:277–313.

3. Negishi K, Negishi T, Kurosawa K, Hristova K, Popescu BA, Vinereanu D, et al. Practical Guidance in Echocardiographic Assessment of Global Longitudinal Strain. JACC Cardiovasc Imaging. 2015;8:489–92.

4. Pieske B, Tschöpe C, De Boer RA, Fraser AG, Anker SD, Donal E, et al. How to diagnose heart failure with preserved ejection fraction: The HFA-PEFF diagnostic algorithm: A consensus recommendation from the Heart Failure Association (HFA) of the European Society of Cardiology (ESC). Eur Heart J. 2019;40:3297–317.
